# Construction and Generation of a Recombinant Senecavirus a Stably Expressing the NanoLuc Luciferase for Quantitative Antiviral Assay

**DOI:** 10.3389/fmicb.2021.745502

**Published:** 2021-10-01

**Authors:** Xiaoran Guo, Kuan Zhao, Xiaona Liu, Baishi Lei, Wuchao Zhang, Xiuli Li, Wanzhe Yuan

**Affiliations:** ^1^College of Animal Medicine, Hebei Agricultural University, Baoding, China; ^2^Hebei Veterinary Biotechnology Innovation Center, Hebei Agricultural University, Baoding, China; ^3^North China Research Center of Animal Epidemic Pathogen Biology, China Agriculture Ministry, Baoding, China

**Keywords:** Senecavirus A (SVA), nanoluciferase, neutralization test, antiviral screening, siRNA

## Abstract

Senecavirus A (SVA), also known as Seneca Valley virus, is a recently emerged picornavirus that can cause swine vesicular disease, posing a great threat to the global swine industry. A recombinant reporter virus (rSVA-Nluc) stably expressing the nanoluciferase (Nluc) gene between SVA 2A and 2B was developed to rapidly detect anti-SVA neutralizing antibodies and establish a high-throughput screen for antiviral agents. This recombinant virus displayed similar growth kinetics as the parental virus and remained stable for more than 10 passages in BHK-21 cells. As a proof-of-concept for its utility for rapid antiviral screening, this reporter virus was used to rapidly quantify anti-SVA neutralizing antibodies in 13 swine sera samples and screen for antiviral agents, including interferons ribavirin and interferon-stimulated genes (ISGs). Subsequently, interfering RNAs targeting different regions of the SVA genome were screened using the reporter virus. This reporter virus (rSVA-Nluc) represents a useful tool for rapid and quantitative screening and evaluation of antivirals against SVA.

## Introduction

Senecavirus A (SVA), formally named Seneca Valley virus, is a single-stranded non-enveloped RNA virus and belongs to the genus Senecavirus of the family Picornaviridae (Hales et al., 2008; Adams et al., 2015). The genome of SVA is about 7.2 kb in length. It contains a unique open reading frame (ORF), flanked by a 5′ untranslated region (UTR) and a short 3′ UTR followed by a poly(A) tail. The ORF is translated into a single polyprotein posttranslationally processed by virus-encoded proteases into the protein products (5′-L-VP4-VP2-VP3-VP1-2A-2B-2C-3A-3B-3C-3D-3′) (Hales et al., 2008). In 2002, a company in the United States accidentally discovered SVA in cell culture (Hales et al., 2008). SVA was originally considered a contaminant in the cell culture, presumably derived from porcine trypsin or fetal bovine serum (Reddy et al., 2007). After 2014, SVA has been associated with vesicular disease outbreaks of swine in Canada, United States, Brazil, Thailand, and China (Vannucci et al., 2015; Joshi et al., 2016; Wu et al., 2017; Xu et al., 2017; Saeng-Chuto et al., 2018). It has been confirmed that pigs infected with SVA can cause porcine vesicular lesions on the snout, oral mucosa, and coronary bands (Montiel et al., 2016). The clinical symptoms of SVA lie in its similarity with another high-consequence vesicular diseases of swine, including foot-and-mouth disease, swine vesicular disease, vesicular stomatitis, and vesicular exanthema of swine (Leme et al., 2015). The public has not recognized many aspects of knowledge related to SVA transmission and pathogenesis. It is urgent to dissect the immunity and pathogenesis and develop vaccines and antivirals.

Reverse genetics is a powerful tool to address the issues with developing recombinant reporter viruses. Inserting foreign tags into the virus genome by reverse genetic manipulation can be used to study the replication of the virus, protein functions, and the screening of antiviral drugs. For example, the marker virus expressing enhanced green fluorescent protein (EGFP) allows rapid detection of viral infection and determination of anti-SVA neutralizing antibodies (NAbs) (Liu et al., 2020a). However, the detection of green fluorescent protein needs the help of a fluorescence microscope, which is highly dependent on subjective judgment and is limited in high-throughput screening (Liu et al., 2020a). Nanoluciferase (Nluc) is a new light-emitting system from deep-sea shrimps (*Oplophorus gracilirostris*) that has been genetically engineered. Not only is the Nluc smaller (∼500 nt) than either firefly or Renilla luciferase but it also has stronger enzymatic activity and maintains longer and more stable luminescence (Hall et al., 2012). In the cases of Nluc-expressing flaviviruses, the enzyme activity was quantified in a simple manner or a high-throughput assay to assess the antiviral activity of potential inhibitors *in vitro* (Brecher et al., 2017).

This study generated a reporter SVA (SVA-Nluc) stably expressing the Nluc gene as an alternative to the EGFP-tagged SVA. The rSVA-Nluc can quickly determine the neutralizing antibody titer of SVA and quantitatively determine the virus proliferation, which can also complete the high-throughput screening of antiviral drugs and molecules.

## Materials and Methods

### Cell, Viruses, Serum, and Antibody

Baby hamster kidney-21 (BHK-21) cells and swine testis (ST) cells were cultured in Dulbecco’s modified Eagle’s medium (DMEM, Gibco, china) at 37°C in a humidified 5% CO_2_ atmosphere. The SVA strain HeB-2019 (GenBank accession number: MZ375462) was the parent virus for generating the reporter virus below. Anti-SVA VP3 monoclonal antibody was kindly provided by Dr. Zhenhai Chen, Yangzhou University, China. Rabbit anti-Flag monoclonal antibody (Cat. no. F7425; 1:5,000) was from Sigma-Aldrich. Goat anti-rabbit IgG (H + L) was from ProteinTech (1:5,000).

### Plasmid, Porcine IFN-α Protein, and Ribavirin

pCAGGS-RIG-I-Flag, pCAGGS-MDA5-Flag, pCAGGS-MOV10-Flag, pCAGGS-ZCCHC3-Flag, pCAGGS-DDX46-Flag, and pCAGGS-Serinc5-Flag, Porcine IFN-α (PoIFN-α) protein were prepared in our laboratory. Ribavirin was purchased from Beijing Solarbio Science and Technology Co., Ltd.

### Construction of a Full-Length Senecavirus A cDNA Infectious Clone Containing the NanoLuc Gene

**FIGURE 1:**
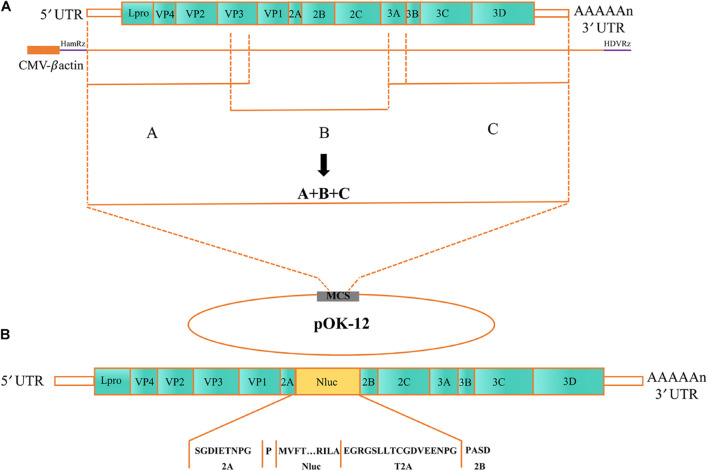
The schematic diagram for the construction of Senecavirus A (SVA)-HeB. **(A)** Schematic diagram of the full-length SVA genome and construction of the full-length cDNA clone. The open reading frames (ORFs) are flanked by 5*′* and 3′ UTR followed by the poly(A) tail at the 3′ end. Three separate genomic fragments (A–C) were synthesized and assemble into the pOK12 vector using the NEBuilder HiFi DNA Assembly Cloning Kit. The full-length viral genome is under the control of a CMV enhancer and β-actin promoter. **(B)** A scheme of the reporter virus genome with a Nluc-T2A fusion gene inserted between SVA 2A and 2B. CMV, cytomegalovirus enhancer; β-actin, beta-chicken actin promoter; HamRbz, hammerhead ribozyme; HDVRz, hepatitis delta virus ribozyme.

To construct an SVA full-length clone, three separate fragments (A, B, and C) were amplified using Q5 High-Fidelity DNA Polymerase (New England BioLabs, Ipswich, MA, United States). The hammerhead ribozyme (HamRbz) element was inserted upstream of fragment A, while a hepatitis D virus (HDV) ribozyme element was fused to the 3′ terminus of the viral genome (fragment C). The three separate fragments are controlled by eukaryotic RNA polymerase II (Pol II), cytomegalovirus (CMV) enhancer, and β-actin promoter. To create a molecular marker for differentiating the cloned virus from the parental virus, a *Mlu*I restriction endonuclease site was introduced with a G5907T synonymous mutation. Subsequently, fragments A, B, and C were assembled into a modified pOK12 vector with NEBuilder HiFi DNA Assembly Cloning Kit (New England BioLabs, Ipswich, MA, United States), and the resulting full-length cDNA clone was designated as rSVA (Figure 1A). The NanoLuc gene (GenBank accession No. MH037010) was synthesized and fused with a Thosea asigna virus 2A element at its C-terminus to construct a full-length cDNA clone expressing NanoLuc. The Nluc-T2A fusion gene was inserted between the SVA genes 2A and 2B using the overlap extension PCR method. The full-length cDNA clone was designated rSVA-Nluc (Figure 1B). BHK-21 cells seeded in a six-well plate were transfected with 3 μg of rSVA-Nluc plasmid using the X-tremeGENE HP DNA Transfection Reagent (Roche, Mannheim, Germany) and were harvested at 72 h post-transfection (hpt), and subjected to three freeze-and-thaw cycles to collect supernatant for serial blind passages in BHK-21 cells. Cytopathic effect (CPE) was monitored daily after infection.

### Identification of rSVA-Nluc

#### Reverse Transcription-PCR and Indirect Immunofluorescence Assay

The culture supernatant of rSVA-Nluc was harvested for extracting viral RNA by TRIzol reagent. For reverse transcription-PCR (RT-PCR), two sets of primer pairs (Table 1) were used: one pair for the Nluc gene (Nluc-F/R) and another pair for the SVA VP3 gene (VP3-F/R). The PCR product was subjected to electrophoresis on a 1% agarose gel and sequenced.

**TABLE 1:** The primers used in the study.

Primers	Sequence (5′ to 3′)
Nluc-F	TAAGCAGAAGATGCTGATGCAAT
Nluc-R	CCAGAGTGACTAAATCGTTTTCCG
VP3-F	TCTCATCTCCTTCCCGATCAC
VP3-R	TGGGCAGTCAGGTGGTAGAGTAAT
F1-F	GTGGGAAGGTATCTTTCGTGCT
R1-R	TCATAGTGGTGAGACTTTGGGC

Baby hamster kidney-21 cells in 48-well plates were infected with rSVA-Nluc. At 24 h, cells were fixed with 4% paraformaldehyde at room temperature for 30 min and permeabilized with 0.1% Triton X-100. Fixed cells were incubated with anti-VP3 monoclonal antibody (1:100 dilution in PBS) for 1 h at 37°C. Subsequently, cells were washed three times with PBS and incubated with FITC-labeled goat anti-mouse IgG (1:200 dilution in PBS) at 37°C for 1 h. After washing three times with PBS, the cells were analyzed under a fluorescence microscope (Carl Zeiss, Germany) with a video documentation system.

#### Virus Growth Kinetics

Replication kinetics of rSVA-HeB or rSVA-Nluc were assessed *in vitro*. BHK-21 cells were infected with both viruses at a multiplicity of infection (MOI) of 0.1 and harvested at various time points post-infection (12, 24, 36, 48, 60, and 72 h post-infection). The viral titers were determined by the Reed–Muench method and expressed as 50% tissue culture infective doses (TCID_50_)/ml. Mean values and standard deviations were calculated from the results from three independent experiments.

#### Nanoluciferase Stability During Passaging

The rescued rSVA-Nluc was serially passaged in BHK-21 cells 10 times. The cell culture supernatants of F1–F10 were harvested to extract viral RNA for RT-PCR analysis using the SVA F1/R1 primer pair (Table 1) to analyze the stability of the foreign sequence in the rSVA-Nluc genome. The RCR reaction underwent 94°C for 5 min, 94°C for 30 s, 58°C for 30 s, 72°C for 1 min, 32 cycles, and 72°C for 10 min. The amplified products were detected by agarose gel electrophoresis, and F5 or F10 were subjected to Sanger sequencing.

#### Nanoluciferase Activity Assay

Time-course analysis of the Nluc expression was performed in 24-well tissue culture plates. BHK-21 cells cultured in 24-well plates were infected with rSVA-HeB or rSVA-Nluc at a multiplicity of infection (MOI) of 0.1. For all assays, the Nluc activity in relative light units (RLU) was measured using the Nano-Glo^®^ Luciferase Assay System (Promega, Madison, WI, United States) with a TD-20/20 luminometer (Turner Designs, Sunnyvale, CA, United States), according to the instructions of the manufacturer.

### Nanoluciferase Activity-Based Neutralization Test

There were 13 swine sera collected from SVA-infection-like pigs stored in our laboratory. The SVA-Nluc-NT was carried out in 96-well plates. Briefly, the sera were heat inactivated for 30 min at 56°C and then were twofold serially diluted (1:8 to 1:1,024) in DMEM and incubated with SVA-Nluc at 37°C for 1 h. The virus-antibody mixtures were transferred to the 96-well plates containing the cell monolayers. The luciferase assay was performed after incubation for 48 h at 37°C, four replicate wells per dilution, with appropriate positive and negative controls. The SVA-specific NAbs titers were expressed as the reciprocal of the highest dilution, resulting in a 50% reduction of the Nluc activity (Shen et al., 2014). In addition, all sera were also tested in parallel by the traditional CPE method.

### Western Blotting and Screening of Antiviral Interferon-Stimulated Genes

Baby hamster kidney-21 cells were seeded in 24-well plates at a cell density of 4 × 10^4^ cells per well. At 14–16 h after plating, cells were transfected with a control plasmid or plasmids expressing RIG-I, MDA5, MOV10, ZCCHC3, DDX46, and Serinc5 (2 μg per well) using X-tremeGENE HP DNA Transfection Reagent. At 24 hpt, cells were infected with rSVA-Nluc at a multiplicity of infection (MOI) of 0.1 for 48 h and assayed for Nluc activity. The screening was run in triplicate. The Nluc activity (rSVA-Nluc) was determined as described above.

### Senecavirus A-Specific Short Interfering RNAs Preparation and Transfection

Six SVA-specific siRNAs (Table 2) targeting the SVA VP1 and 3D genes (three siRNA for each gene) were designed based on the genome of the SVA and a control scramble siRNA, which has no matches either in the viral. All siRNAs were synthesized by Shanghai GenePharma.

**TABLE 2:** Sequence of siRNAs test in this study.

Target genes	Names of siRNAs	Sense (5′ to 3′)	Antisense (5′ to 3′)
VP1	SVA VP1-sus-37	GGUAACACUGACACCGAUUTT	AAUCGGUGUCAGUGUUACCTT
	SVA VP1-sus-295	GGCGUUCUCGCUAAUACUUTT	AAGUAUUAGCGAGAACGCCTT
	SVA VP1-sus-340	GCCUGUUUCACUUACUUUATT	UAAAGUAAGUGAAACAGGCTT
3D	SVA 3D-sus-81	GGUGUACAAACCGGAGUUUTT	AAACUCCGGUUUGUACACCTT
	SVA 3D-sus-417	GGCCAUGCAAAUCCAGAAATT	UUUCUGGAUUUGCAUGGCCTT
	SVA 3D-sus-949	GCAUUGACCUACAAGGAAUTT	AUUCCUUGUAGGUCAAUGCTT

When cell monolayers were 80% confluent, siRNA was introduced using X-tremeGENE siRNA Transfection Reagent (Roche, Mannheim, Germany) according to the protocol of the manufacturer, with an siRNA final with a concentration of 100 nM. After incubation for 24 h, rSVA-Nluc at an MOI of 0.1 was added to the cells. At 48 h, the Nluc activity and the viral titers were determined as described above.

### Evaluation of Antiviral Effects of Ribavirin and Porcine IFN-α Based on rSVA-Nluc

Baby hamster kidney-21 cell monolayers were pretreated with various concentrations of ribavirin (0, 5, 10, 20, 50, 100, and 200 μM), five replicate wells per dilution. The ribavirin-treated cells were incubated at 37°C for 24 h. Subsequently, MTT [3-(4,5-dimethylthiazol-2-yl)-2,5-diphenyltetrazolium bromide] assay (Takeuchi et al., 1991) is used to detect cytotoxicity. Based on the result of the cytotoxicity assay, the cells were infected with the rSVA-Nluc at an MOI of 0.1 for 1 h and were subsequently treated with the same Ribavirin concentration. At 48 h, the Nluc activity (rSVA-Nluc) was determined as described above.

The anti-SVA infection activities of the PoIFN-α protein were detected with the cytopathic effect inhibition assay (Favalli et al., 1985) in ST cells. Briefly, ST cells were cultured in DMEM supplemented with 10% fetal bovine serum in a 96-well plate and treated with 100 μl of fourfold serial dilutions of PoIFN-α for 24 h at 37°C. After 16 h, cells were inoculated with 100 μl of SVA-Nluc (100TCID_50_), and also the positive control and negative control were included. At 48 h, the cytopathic effect was observed under a microscope. Then the Nluc activity (rSVA-Nluc) was determined as described above.

### Statistical Analysis

All experiments mentioned above were performed with three independent experiments. The GraphPad Prism software was used for statistical analysis by a two-tailed Student’s *t*-test. The data shown are the means ± standard variations (SD) of three independent experiments.

## Results

### Generation of the Recombinant SVA-Nluc

The overall strategy to construct the infection clone of the SVA strain HeB is shown in Figure 1A. Fragments A, B, and C were synthesized and assembled (see section “Materials and Methods”). The full-length genome sequence of SVA was then engineered into the pOK12 vector, producing the rSVA. The backbone of the rSVA-Nluc cDNA clone was the rSVA cDNA clone. As shown in Figure 1B, the Nluc-T2A fusion sequence was inserted between the 2A and 2B sequences of the rSVA cDNA clone (see section “Materials and Methods”). The full-length cDNA clone was designated as rSVA-Nluc.

**FIGURE 2:**
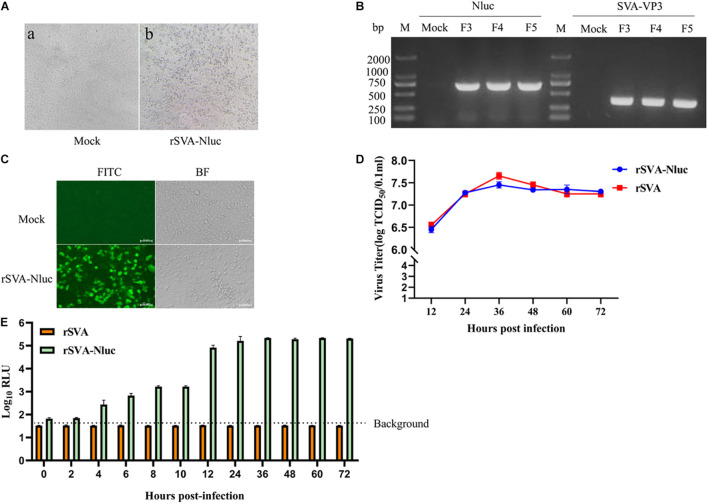
Characterization of the SVA-Nluc. **(A)** Cytopathic effect (CPE) of the rSVA-Nluc cloned viruses in BHK-21 cells; Mock-infected cells were used as the negative control. **(B)** RT-PCR analysis of the Nluc gene and SVA VP3 gene at F3, F4, and F5 using Nluc-F/R and the VP3-F/R primer pairs. **(C)** Immunofluorescence assay (IFA) of the rSVA-Nluc in BHK-21 cells and mock control without virus infection. BHK-21 cells were infected with the rSVA-Nluc in 48-well plates at a multiplicity of infection (MOI) of 0.5 and analyzed using the indirect immunofluorescent assay at 24 h post-infection. **(D)** Multiple-step virus growth curve. BHK-21 were infected rSVA-HeB or rSVA-Nluc at an MOI of 0.5. Viral titers from culture supernatants at indicated time points were determined by the Reed–Muench method and expressed as 50% tissue culture infective doses (TCID_50_)/ml. Each data point shown represents the mean value from duplicates, and error bars show standard errors of the mean (SEM). **(E)** Time-course analysis of the nanoluciferase reporter gene expression. BHK-21 cells cultured in 24-well plates were infected with rSVA-HeB or rSVA-Nluc at an MOI of 0.5 and assayed for the Nluc activity in relative light units (RLU) at the indicated time points. Data represent mean values of three independent experiments with error bars representing the standard deviations of the means.

The plasmid rSVA-Nluc was transfected into BHK-21 cells to rescue the rSVA-Nluc virus. After 15 h, the BHK-21 cells showed typical CPE compared with the negative control groups (Figure 2A). Total RNA was extracted from the F3 to F5 rSVA-Nluc and analyzed by RT-PCR. The results showed that the 677-bp product of the Nluc and 379-bp product of the VP3 were successfully amplified from all viruses (F3–F5) (Figure 2B). What is more, the immunofluorescence showed that a strong positive signal could be detected in the cells infected with the rSVA-Nluc, indicating that the rSVA-Nluc was successfully rescued from the BHK-21 cells (Figure 2C).

### Replication Properties of the Recombinant Virus and Stability of Nanoluciferase

**FIGURE 3:**
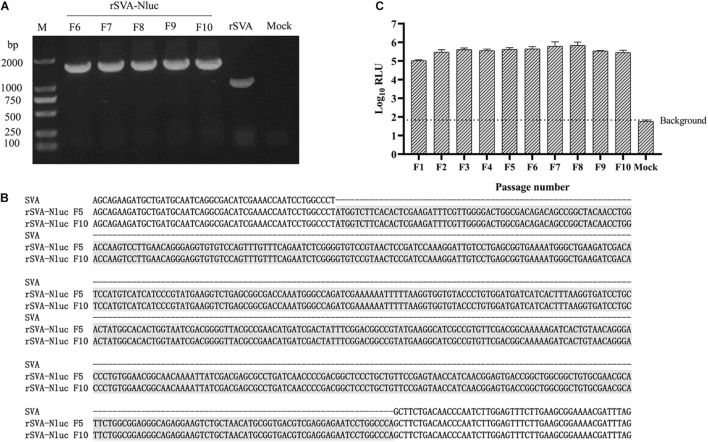
Stability of the Nluc expressed by the rSVA-Nluc during serial passaging in BHK-21 cells. **(A)** RT-PCR analysis of the Nluc gene during virus passage. Viral RNA was extracted from culture supernatants of different passages in BHK-21 cells, and RT-PCR was performed with F1/R1 primer pair. **(B)** Sequence alignment analysis to assess the genetic stability of the F5 and F10 recombinant viruses. **(C)** The Nluc activity of rSVA-Nluc of F1-F10 passage. BHK-21 cells cultured were infected with rSVA-Nluc at an MOI of 0.5 and assayed for the Nluc activity in relative light units (RLU) at 48 h post-infection. Data represent mean values of three independent experiments with error bars representing the standard deviations of the means.

The growth kinetics of the rSVA-Nluc virus and parental virus rSVA were compared to determine whether the expression of Nluc affected virus replication. The result indicated that the rescued SVA-Nluc shared similar replication kinetics with its parental virus passaged in BHK-21 cells (Figure 2D). The Nluc activity of SVA-Nluc was measured at different time points (0, 2, 4, 6, 8, 10, 12, 24, 36, 48, 60, and 72 h). The results suggested that Nluc activity of SVA-Nluc-infected cells can be detected at 4 hpi, and the Nluc activity increased to a peak at 36 hpi, approximately 6,476-fold higher than the rSVA-infected group (Figure 2E). The culture supernatant of passages 1–10 virus was harvested for detecting the rSVA-Nluc fusion gene by RT-PCR to determine the stability of the Nluc gene at the insertion site. The RT-PCR products with an expected 1,667 bp were detected from the supernatant of the infected BHK-21 cells (Figure 3A). After nucleotide sequencing of the exogenous luciferase gene inserted region, the sequence alignment data showed no mutant sites in the region of interest. The results confirmed the existence of intact Nluc in rSVA-Nluc (Figure 3B). We also measured the Nluc activity of F1–F10. The luciferase activity of the rSVA-Nluc did not decrease by the subsequent cell passages (Figure 3C).

### A Rapid Neutralization Assay for Senecavirus A-Positive Sera

**FIGURE 4:**
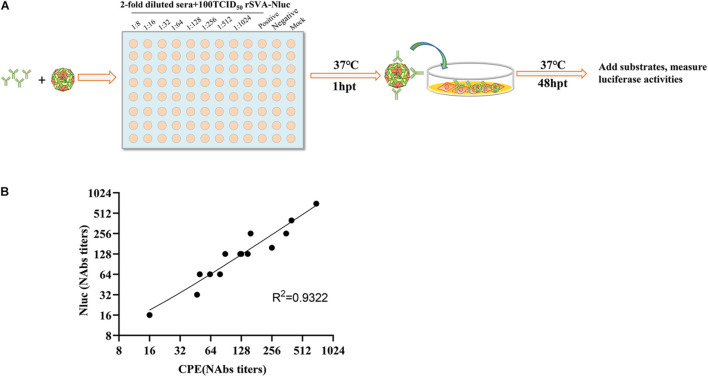
Correlation between SVA-Nluc and traditional PRNT50 assays. **(A)** A schematic of the rapid neutralization assay. **(B)** The relationship between the traditional PRNT50 assays and the logarithms of the Nabs titers was analyzed based on the data from 13 serum samples.

We tested 13 SVA-positive sera based on rSVA-Nluc. Nluc signals were measured to determine the serum dilution that neutralized 50% of Nluc activity (NT50). Figure 4A depicts the flowchart of the rSVA-Nluc neutralization assay in a 96-well format. To validate the Nluc neutralization results, we performed neutralizing titers against serotype (PRNT50) on the same set of positive sera. The neutralization results between the Nluc virus and traditional PRNT50 assays had a correlation coefficient (*R*^2^) of 0.93 (Figure 4B). The results showed that the rSVA-Nluc neutralization assay detecting neutralizing antibodies in SVA sera had a higher sensitivity than the conventional CPE assay.

### Screening of Interferon-Stimulated Genes and Short Interfering RNA for the Ability to Inhibit Senecavirus A Replication

**FIGURE 5:**
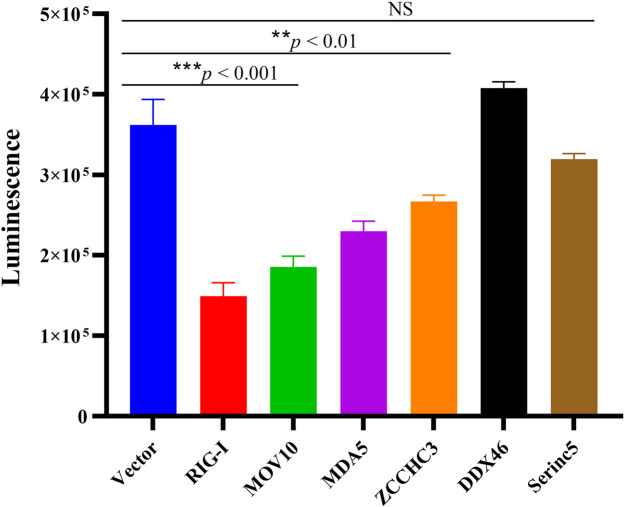
Screening of ISGs using rSVA-Nluc infection. BHK-21 cells were transfected with ISGs followed by infection with rSVA-Nluc at an MOI of 0.1 for 48 h and assayed for Nluc activity. The data are presented as the mean ± SD from three experiments. The statistical significance of differences was determined using Student’s *t*-test (NS, not significant; *p < 0.05; **p < 0.01; ***p < 0.001).

To further evaluate the applicability of rSVA-Nluc for antiviral ISG screening, the pCAGGS-RIG-I-Flag, pCAGGS-MDA5-Flag, pCAGGS-MOV10-Flag, pCAGGS-ZCCHC3-Flag, pCAGGS-DDX46-Flag, and pCAGGS-Serinc5-Flag were confirmed with Western blotting (data not shown; the WB results are available upon request). Seven recombinant plasmids were, respectively, transfected into BHK-21 cells, then infected with rSVA-Nluc at 0.5 MOI for 16 h. As expected, the Nluc activity was significantly reduced by RIG-I. Notably, overexpression of MDA5, MOV10, or ZCCHC3 significantly decreased Nluc activity in rSVA-Nluc-infected cells, while overexpression of DDX46 or Serinc5 is similar to the control group (Figure 5).

**FIGURE 6:**
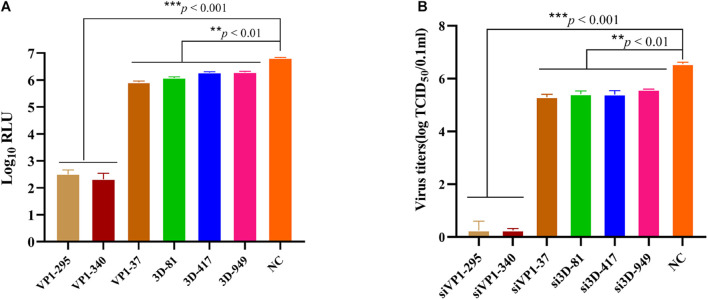
Antiviral siRNAs screening using rSVA-Nluc. **(A)** Screening of antiviral siRNAs using rSVA-Nluc. BHK-21 cells were transfected with six siRNAs followed by infection with rSVA-Nluc at an MOI of 0.1 for 48 h and assayed for Nluc activity. **(B)** Viral titers of rSVA-Nluc in siRNA-treated cells. BHK-21 cells were transfected with six siRNAs followed by infection with rSVA-Nluc at an MOI of 0.1 for 48 h. TCID_50_ values are the means of three repeat titrations at the time points indicated. Data represent three independent experiments and are shown as mean ± standard deviation, and *p*-values of paired Student’s *t*-test are shown (NS, not significant; *p < 0.05; **p < 0.01; ***p < 0.001).

SVA-specific siRNAs targeting VP1 and 3D were evaluated for anti-SVA-Nluc activities in BHK-21 cells. The Nluc activity was significantly decreased in the cells transfected with any siRNAs at a concentration of 100 nM, especially siVP1-295 and siVP1-340 (Figure 6A). As measured by TCID50 assay, the viral titer was significantly decreased in cells transfected with any siRNAs (Figure 6B). The results demonstrated the feasibility of using rSVA-Nluc for antiviral screening.

### A High-Throughput Antiviral Assay for Senecavirus A

**FIGURE 7:**
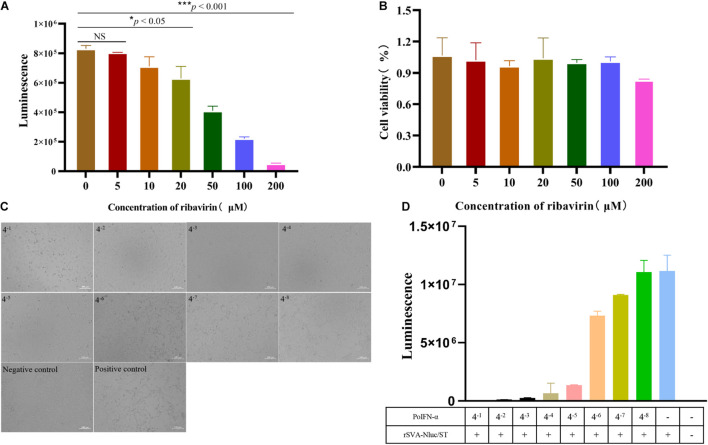
Screening of antiviral drug and PoIFN-α protein using rSVA-Nluc. **(A)** Effects of ribavirin on rSVA-Nluc replication. BHK-21 cell monolayers were pretreated with various concentrations of ribavirin followed by infection with rSVA-Nluc at an MOI of 0.1 in 48-well plates, and the Nluc activity was measured at 48-h post-infection. **(B)** MTT [3-(4,5-dimethylthiazol-2-yl)-2,5-diphenyltetrazolium bromide] method is used to detect BHK-21 cell cytotoxicity. The statistical significance of differences was determined using Student’s *t*-test (NS, not significant; *p < 0.05; **p < 0.01; ***p < 0.001). **(C)** The effect of different concentrations of PoIFN-α on SVA-infected cell pathology. ST cell monolayers were pretreated with various concentrations of PoIFN-α followed by infection with rSVA-Nluc at an MOI of 0.1 in 96-well plates, the cytopathic effect was observed under a microscope. **(D)** Luciferase activity to detect the effect of PoIFN-α on virus replication.

Reporter viruses have been commonly used for antiviral screening. Therefore, we developed a 96-well format antiviral assay using the rSVA-Nluc reporter virus. There are several reports that ribavirin can effectively inhibit SVA replication at the cellular level (Li C. et al., 2019; Liu et al., 2020a). Thus, ribavirin was used to evaluate the assay in BHK-21 cells. As expected, Nluc activity was reduced in the presence of increasing levels of ribavirin in a dose-dependent manner (Figure 7A). The effects of drugs on BHK-21 cell viability were determined by the MTT assay. The ribavirin concentrations were not greatly toxic to the cells (Figure 7B).

PoIFN-α proteins were tested for anti-SVA-Nluc activities in ST cells. The CPE results showed that as the dilution of PoIFN-α protein increases, the CPE becomes more and more obvious, which is dose dependent (When the dilution of PoIFN-α is 4^–1^, it will damage the cells) (Figure 7C). The Nluc activity was increased in the presence of increasing dilution of IFN-α protein in a dose-dependent manner (Figure 7D). The above results indicated that the recombinant rSVA-Nluc could be used as a tool for screening SVA virus drugs due to its high throughput and high sensitivity.

## Discussion

In recent years, SVA has demonstrated its capacity to cause vesicular diseases and neonatal mortality in pigs, and the increasing incidence of SVA infection in pigs will continue to cause unpredictable and substantial outbreaks. Although some progress has been achieved in the study of SVA, many aspects of knowledge such as antivirals against SVA, vaccines available, and the antiviral innate immunity of the host remains highly lacking. The goals of this study were to (i) develop a rapid neutralization assay, (ii) establish a high-throughput assay for reliable antiviral screening. We established a nanoluciferase SVA (rSVA-Nluc) as a platform for rapid serodiagnosis and high-throughput drug screening.

Viruses containing reporter genes within their genomes are a useful tool for detection and/or quantification of viral replication and screening antiviral agents. In recent years, the SVA reverse genetic systems have been widely used to construct recombinant viruses to express foreign genes (Liu et al., 2020a, 2021; Wang et al., 2021). A high-throughput neutralization test and screening of antiviral drugs based on an EGFP reporter SVA virus have been established to measure Nabs and antiviral drugs (Liu et al., 2020a). However, EGFP-based detection methods require fluorescence microscopy for judgment and are unsuitable for high-throughput screening (HTS) detection methods. In this study, we developed a stable reporter SVA-Nluc recombinant virus. As a novel engineered product, compared with firefly and Renilla luciferases, the nanoluciferase tag has several advantages. For example, first, enhanced stability, smaller size, and >150-fold increase in luminescence. Second, it can be used in HTS assays in 96-well plates, screening several hundred compounds of antivirals. Third, it is time saving and more sensitive (Shen et al., 2014; England et al., 2016). That is why the Nluc system is widely used in rapid neutralization testing and high-throughput antiviral drug screening among various viruses (Wang et al., 2019; Baker et al., 2020; Xie et al., 2020; Yao et al., 2021).

We developed a stable reporter SVA-Nluc recombinant virus for rapid neutralization testing. Since neutralizing titer is a key parameter to detect immunity, the rapid SVA-Nluc neutralization assay will enable many aspects of SVA research, including laboratory diagnosis, vaccine development, antiviral study, etc. The neutralizing antibody titers derived from the reporter virus assay were equivalent to those derived from the conventional PRNT50 assay (Figure 4B). The rSVA-Nluc-NT provides several significant advantages over the conventional PRNT50 assay and rSVA-eGFP-based VNT. First, rSVA-Nluc-NT can be used in high-throughput neutralizing antibody assay. Although the current detection is performed in a 96-well format, considering the amplitude and dynamic range of the Nluc signal, it can be easily adapted to the 384-well format. Moreover, the sample plates can be frozen and stored (after cell lysis) for several days without affecting the read-outs. Second, due to the amplification nature of the Nluc enzyme, the rSVA-Nluc has a larger dynamic range and higher sensitivity than the rSVA-eGFP virus assay (Liu et al., 2020a). We have shown that this system is valuable for rapidly testing sera for neutralizing antibodies and compounds for antiviral activity.

Besides, reporter viruses have been used widely for screening ISGs and siRNAs to discover host factors that can influence the replication of viruses (Karlas et al., 2010; Shen et al., 2014; Li et al., 2016). RIG-I presented an antiviral role against SVA and was essential for activating type I IFN signaling during SVA infection (Li et al., 2018). To verify whether the Nluc activity of rSVA-Nluc could be used for ISG screening, RIG-I as a positive control, using a luciferase-based ISG screening assay, we identified two host factors (MOV10 and ZCCHC3) that exhibit antiviral effects. MOV10 gene belongs to the UPF-1-like helicase superfamily 1 (SF1) and exhibits ATP-dependent 5′ to 3′ helicase activity (Li J. et al., 2019). It is an important host antiviral factor. MOV10 can inhibit various viruses, including human immunodeficiency virus type 1, hepatitis C virus, and influenza A virus (Li J. et al., 2019; Liu et al., 2019). Besides, ZCCHC3 is a co-receptor for RIG-I and MDA5, which is critical for RLR-mediated innate immune response to RNA virus; ZCCHC3 deficiency markedly inhibited RNA virus-triggered induction of downstream antiviral genes, and ZCCHC3-deficient mice were more susceptible to RNA virus infection (Lian et al., 2018). Therefore, MOV10 or ZCCHC3 can inhibit SVA replication, but its molecular mechanism needs further study. RNAi is triggered by small non-coding RNA molecules (ncRNAs), including short interfering RNAs (siRNA) and microRNAs (miRNA), and the use of RNAi-based methods have been demonstrated as an alternative method of controlling the transmission of various viruses (Du et al., 2011; Shi et al., 2015; Matsui et al., 2019; Bai et al., 2020). To the best of our knowledge, no siRNAs targeting VP1 and 3D of SVA have been reported. Here, we assessed 6 siRNA against the SVA VP1 or 3D genes using SVA-Nluc with two siRNAs (siVP1-340 or siVP1-295) to inhibit the SVA-Nluc replication efficiently.

Traditional TCID_50_ assays are ill suited for HTS assays, as more and more novel antiviral drugs are developed every year. To solve this problem, based on Fluc-tagged viruses have been widely used for antiviral screening (Shen et al., 2016; Liu et al., 2020b). Ribavirin is a common anti-picornavirus drug with low cytotoxicity (Choi et al., 2018). IFN has antiviral, anti-tumor, and immune regulation activities (Kalliolias and Ivashkiv, 2010). Thus, we constructed the rSVA-Nluc recombinant virus to assess the effects of ribavirin and PoIFN-α protein on SVA replication. The results showed that the Nluc activity was reduced in the presence of increasing levels of an antiviral drug in a dose-dependent manner (Figures 7A,D). To our knowledge, this is the first report that a Nluc reporter SVA was constructed and used for antiviral drug testing. rSVA-Nluc represents a superior model for screening broad-spectrum SVA drugs without the need to observe CPE lesions to determine the inhibitory effect of antiviral drugs on SVA, which saves time and effort.

In summary, we generated a stable Nluc-based recombinant SVA. Its Nluc activity characteristics made the rSVA-Nluc-based TCID50 assay faster and more accurate than the conventional method, thus, having a great potential in VNT. The reporter virus can be used for rapid and sensitive screening of anti-SVA drugs and host factors. We believe that reporter virus tools are useful for studying SVA pathogenesis and vaccine development in the future.

## Data Availability Statement

The raw data supporting the conclusions of this article will be made available by the authors, without undue reservation.

## Author Contributions

WY, XG, and KZ designed the experiments. XG, KZ, XiaL, BL, WZ, and XiuL performed the experiments. XG and KZ analyzed the data and wrote the manuscript. All authors contributed to the article and approved the submitted version.

## Conflict of Interest

The authors declare that the research was conducted in the absence of any commercial or financial relationships that could be construed as a potential conflict of interest.

## Publisher’s Note

All claims expressed in this article are solely those of the authors and do not necessarily represent those of their affiliated organizations, or those of the publisher, the editors and the reviewers. Any product that may be evaluated in this article, or claim that may be made by its manufacturer, is not guaranteed or endorsed by the publisher.
